# A Comprehensive Review of Intranasal Insulin and Its Effect on the Cognitive Function of Diabetics

**DOI:** 10.7759/cureus.17219

**Published:** 2021-08-16

**Authors:** Mrunanjali Gaddam, Abhishek Singh, Nidhi Jain, Chaithanya Avanthika, Sharan Jhaveri, Ivonne De la Hoz, Sujana Sanka, Sri Rupa Goli

**Affiliations:** 1 Internal Medicine, Andhra Medical College, Visakhapatnam, IND; 2 Internal Medicine, Himalayan Hospital, Dehradun, IND; 3 Internal Medicine, Sir Ganga Ram Hospital, New Delhi, IND; 4 Pediatrics, Karnataka Institute of Medical Sciences, Hubli, IND; 5 Internal Medicine, Smt. Nathiba Hargovandas Lakhmichand Municipal Medical College, Ahmedabad, IND; 6 Internal Medicine, Universidad del Zulia, Maracaibo, VEN; 7 Internal Medicine, JC Medical Center, Orlando, USA; 8 Internal Medicine, Shri Sathya Sai Medical College and Research Institute, Chennai, IND

**Keywords:** intranasal insulin, alzheimer’s disease, diabetes mellitus, cognitive functions, type 2 diabetes mellitus, type 1 diabetes mellitus, mild cognitive impairment, intranasal administration, hyperglycemia, mitochondrial biogenesis

## Abstract

Diabetes mellitus continues to be a disease that affects a good percentage of our population. The majority affected need insulin on a day-to-day basis. Before the invention of the first manufactured insulin in 1978, dealing with diabetes took a significant toll on patient's lives. As technology and human innovation prevail, significant advancements have taken place in managing this chronic disease. Patients have an option to decide their mode of insulin delivery. Intranasal insulin, one such form, has a rapid mode of action while effectively controlling postprandial hyperglycemia. It has also been proven to reduce hypoglycemia and insulin resistance problems, which seem to be the main adverse effects of using conventional insulin regularly. However, due to the large dosages needed and high incurring costs, Intranasal Insulin is currently being used as adjunctive therapy along with conventional insulin.

We conducted a literature search in PubMed indexed journals using the medical terms "Intranasal insulin," "diabetes," and "cognitive impairment" to provide an overview of the mechanism of action of Intranasal Insulin, its distinctive cognitive benefits, and how it can be compared to the standard parenteral insulin therapy. One unique feature of intranasal insulin is its ability to directly affect the central nervous system, bypassing the blood-brain barrier. Not only does this help in reducing the peripheral side effects of insulin, but it has also proven to play a role in improving the cognitive function of diabetics, especially those who have Alzheimer's or mild cognitive impairment, as decreased levels of insulin in the brain has been shown to impact cognitive function negatively. However, it does come with its limitations of poor absorption through the nasal mucosa due to mucociliary clearance and proteolytic enzymes, our body's natural defence mechanisms. This review focuses on the efficacy of intranasal insulin, its potential benefits, limitations, and role in cognitive improvement in people with diabetes with pre-existing cognitive impairment.

## Introduction and background

Insulin is a peptide hormone secreted by the pancreas, specifically by the β cells of Langerhans' islets that maintain normal blood glucose levels by mediating cellular glucose uptake and regulating carbohydrate, lipid, and protein metabolism [1]. In 1922, it was first used as a therapeutic alternative in patients with diabetes, and since then, it has become the cornerstone of treating patients with type 1 diabetes mellitus (T1DM). It is also widely used in patients with type 2 diabetes mellitus (T2DM). However, one of the biggest challenges in insulin therapy is that the injectable route constitutes the most effective and most commonly used option, leading to patient pain and discomfort that negatively impacts medication compliance. Besides, the complex schedules patients have to follow to mimic the physiologic secretion of insulin represent a vital obstacle that still needs to be tackled. To this end, other administration routes have been explored including, transdermal, pulmonary, ocular, nasal, among others [2].

Overall, the intranasal route shows the most convenient profile since it allows the hormone to be delivered directly to the central nervous system [3]. The brain, once considered an insulin-insensitive organ, is currently conceived as an essential insulin target. However, the exact role insulin plays among the different brain structures looks pretty complex and poorly understood. It is known that insulin receptors can be found in significant amounts in the olfactory bulb, hypothalamus, and cerebellum and areas involved in memory, including the hippocampus and limbic system. Additionally, central nervous insulin signalling has been demonstrated to participate in various functions such as peripheral energy and glucose homeostasis, food intake, growth, and neuronal plasticity [4,5]. Intranasal insulin administration represents an efficient instrument to understand better the actual contribution insulin makes to these functions and their potential therapeutic targets [6].

Over the last few years, it has been confirmed that there is a significant association between Diabetes Mellitus and cognitive impairment. This affiliation is considered to be more assertive in type 2 than type 1 diabetes. People with diabetes have a greater rate of decline in cognitive function with a 1.5-fold greater risk of accelerated cognitive decline and a 1.6-fold greater risk for the development of future dementia makes this relationship irrefutable [4,7]. Globally, approximately 422 million patients with T2DM and 25% are older than 65 years [8,9]. Therefore, diabetes-related cognitive dysfunction can seriously challenge the demand for future health resources because of the increasing prevalence and prolonged lifespan [10].

Possible pathophysiologic mechanisms behind this phenomenon include impaired insulin signalling or insulin resistance in the brain [5,10]. In addition, lower insulin levels in the cerebrospinal fluid and a reduced expression of insulin receptors throughout the central nervous system have been found in patients with Alzheimer's Disease [4]. As a result, Intranasal insulin started to draw attention as a potential alternative for patients with different degrees of memory decay and has become an attractive option in Alzheimer's research. Several studies favour its use in humans after demonstrating improved cognition with negligible adverse effects [11]. Nevertheless, the mechanisms for insulin-related improvement of memory (hippocampal function) remain unclear. The long-term effects of intranasal insulin on cognition in diabetic and non-diabetic adults require further research to be unravelled [6].

## Review

Mechanism of action

Intranasal (IN) administration of insulin helps in controlling hyperglycemia. Further, it also reported improvement of cognitive function, human memory and prevented cerebral atrophy [12]. 

After administering IN insulin, it reaches the brain either by the olfactory and trigeminal pathway through the cribriform plate or attaches at specific receptors (insulin receptors) in the blood-brain barrier and then reaches the brain [12,13]. Insulin receptors are distributed throughout the brain, specifically in the olfactory bulb, cerebral cortex, cerebellum, amygdala, hippocampus, and septum [14]. 

The insulin receptor (IR) is a tyrosine kinase receptor and transmembrane protein. IR consists of two extracellular alpha(∝) and two transmembrane beta (β) units. It functions as an allosteric enzyme, where the alpha(∝) subunit prevents the tyrosine kinase activity of the beta(β) subunit [15]. Downstream signalling happens via two pathways- phosphoinositide-3 Kinase (PI3K) and mitogen-activated protein kinase (MAPK) [15].

When IN insulin is given, it binds onto the IR, and autophosphorylation of IR occurs. Hence, it leads to the tyrosine phosphorylation of insulin receptor substrate (IRS) and activates the PI3K and MAPK pathways. APPL1 is an adaptor protein that helps interact with IR and IRS and further activates PI3K and MAPK pathways [15]. These pathways also help produce hypoxia-inducible factor-1 (HIF1) and help in brain angiogenesis through the proliferation of endothelial cells and pericytes [15]. 

Hippocampus has a high concentration of insulin receptors and helps maintain declarative memory, which can be stored for long and recollected [14,16]. Synaptic plasticity means acquiring and maintaining memories, and intranasal insulin helps maintain synaptic plasticity in the hippocampus. Another area of interest in recent literature is the psychological impact of IN insulin. Studies have reported that administration of IN insulin for eight weeks had an antidepressant and anxiolytic impact on study subjects [16]. 

It's been reported that deprivation of insulin in the brain decreased the production of mitochondrial fusion proteins (mitofusin1 (MFN1), mitofusin2 (MFN2), and optic atrophy protein(OPA1)) and increased the production of fission proteins (dynamite related protein(DRP1)). Hence, the mitochondrial function will be impaired, and the cerebrum, hypothalamus, and hippocampus, rich in insulin receptors, will be compromised. Hence, IN insulin is advised to improve memory and cognitive function in diabetics [17].

Pharmacokinetics and Pharmacodynamics

Some drugs, particularly those involving peptide and protein hormone replacement or supplementation, such as insulin, benefit from nasal administration. Several studies have looked at the pharmacokinetics and pharmacodynamics of intranasal insulin.

Leary et al. did an exploratory study to look into a unique insulin formulation for intranasal administration that used CPE-215 technology. They used eight healthy fasting volunteers who were given up to four doses of intranasal insulin spaced at least one week apart in the trial. They discovered that at doses of 18.75 IU and higher, serum insulin levels increased while plasma glucose and serum C-peptide levels decreased. Insulin levels peaked in 10-20 minutes and remained elevated for about an hour, while the resulting drop in glucose peaked at 40 minutes and then returned to an average of 1.5-2 hours after the dose. The average decrease in plasma glucose was 20.48 per cent at 25 IU. Higher insulin doses resulted in a more significant drop in plasma glucose [18].

Following the exploratory trial, Leary et al. conducted a preliminary study in individuals with type 1 diabetes to investigate unique intranasal insulin formulation parameters using subcutaneous insulin or placebo as a comparative. They tested seven fasting volunteers who were given up to four doses of medicine (placebo, subcutaneous insulin, or intranasal insulin) separated by at least three days. According to the findings, the relative bioavailability of IN insulin compared to SC insulin was 16.6-19.8% over 2 hours and 14.0-19.8% over 5 hours. The IN Insulin formulation was well tolerated, with an increase in serum insulin levels and a drop in plasma glucose at dosages of 25 IU and higher. According to the study, peak insulin levels were frequently reached in 15-20 minutes and stayed elevated for around an hour. The impact on glucose peaked at 40 minutes and faded 1.5 to 2 hours after the dose [19].

In another trial, thirteen fasting healthy male volunteers were given five doses of medicine (one dosage of 4 international units [IU] subcutaneous (SC) conventional insulin and four doses of 25 IU IN insulin) taken at least 48 hours apart. Their findings revealed a rise in serum insulin levels accompanied by a drop in plasma glucose, with the highest insulin levels being reached in 10-20 minutes. For 40-50 minutes, the insulin levels remained elevated. With its subsequent effect on glucose levels, this elevation in insulin levels peaked at 40 minutes and then faded about 2 hours after the dose. There was also a statistically insignificant difference in the comparative absorption of IN insulin vs SC insulin according to the nostril status [dominant: 12.0%; nondominant: 15.4%]. Finally, the study found that IN insulin was well tolerated and absorbed rather effectively [20].

Intranasal insulin-containing phospholipid as an absorption enhancer has also been explored in terms of pharmacokinetics. Drejer K et al. tested eleven fasting volunteers who were given 4 U insulin intravenously, 6 U subcutaneously, or three doses intranasally in random order on five different days. The absorption of intranasal insulin was dose-dependent, with a mean plasma insulin peak 16-30 minutes after delivery. The mean plasma glucose nadir was observed around 38-50 minutes, about 20 minutes after intravenous infusion. These findings were consistent with the findings of the other research in that there is minor intersubject variation (p <0.01) [21].

One study comparing the pharmacokinetics of two concentrations of a proprietary formulation of an ultra rapid-acting IN insulin formulation [Nasulin] found a proportional and linear dose-response for pharmacokinetics and pharmacodynamic parameters, implying that multiple doses could be made available for clinical development [22].

To summarise, intranasally administered insulin has a remarkably exact absorption and impact timing, resulting in almost identical insulin and physiological profiles. There have only been a few long-term studies published which have been compared in Table 1. Because of its rapid absorption and beginning of an action, intranasal insulin is equally effective as standard subcutaneous insulin in reducing postprandial hyperglycemia excursions. The tendency to cause postprandial hypoglycemia is like subcutaneous therapy and more closely reflects physiological meal-induced insulin. It is necessary to address concerns concerning the long-term usage of intranasally administered insulin. These include its limited absorption, and as a result, the significant amount of insulin required. However, recombinant deoxyribonucleic acid technology may solve this problem in the future by increasing availability and lowering the cost of insulin. The reproducibility and effect of long-term treatment, as well as the possibility of nasal mucosa irritation, must be examined further. In addition, we must scrutinize the impact of a common cold or a congested nose on insulin absorption [23].

**Table 1:** A comparative table of the different studies conducted on intranasal insulin and the common parameters measure

Study	Study population	Peak insulin level achieved after	Average decrease in plasma glucose	Relative bioavailability
Leary et al. [18]	Eight fasting healthy volunteers (four men, four women; body mass index, 23.6 ± 1.7 kg/m^2^)	10–20 min	20.49%	10–15%
Leary et al. [19]	Seven fasting volunteer patients with type 1 diabetes (five men, two women; body mass index 23.54 ± 1.32 kg/m^2^)	15–20 min	20.5%	16.6–19.8% over 2 h and 14.0–19.8% over 5 h
Leary et al. [20]	Thirteen fasting healthy male volunteers received five doses of medication (one dose of 4 international units [IU] subcutaneous (SC) regular insulin and four doses of 25 IU IN insulin) at least 48 h apart	10–20 min	20.5%	15–20%
Drejer et al. [21]	Eleven fasting subjects received 4 U insulin intravenously, 6 U subcutaneously, or three doses intranasally (approximately 0.3 U kg-', 0.6 U kg-', 0.8 U kg-') in random order on five separate days	23 min	20%	16.9 - 23.9%
Stote et al. [22]	24 healthy, nonsmoking subjects (ages 18–50, basal metabolic index 70 kg)	10–20 min		

Intranasal insulin and its efficacy in DM

Insulin has been in use for the treatment of DM for nearly 100 years now. During this time, due to rigorous research and development, everything from the delivery system to the molecule itself has undergone a drastic change.

The cornerstone of conventional insulin therapy is the subcutaneous method, which requires single or many daily injections [24]. Despite the introduction of user-friendly insulin pens, injectable therapy does not hold the best patient compliance [25]. In addition, despite constancy in the parenteral dose regimen and injection site, metabolic variability in glycemic control exists in most insulin-dependent diabetics. Efforts today are being made to design insulin delivery systems that replicate the physiological release of insulin in the body, an advantage thus offered by the intranasal delivery system [2].

A robust selection of oral and injectable hypoglycemics and insulin analogues are already available. Intranasal insulin, other than the cognitive benefits, offers slight advantages.

In type 2 diabetics, it was found that intranasal insulin did not only affect fasting and postprandial blood glucose levels but did so in a way that simplified dosing too [26]. Frauman et al. showed that a single intranasal dose of 30 units of porcelain insulin was an effective alternative to oral hypoglycemics, avoiding many of the issues that come with their use in clinical practice [27]. Moreover, a second dose may be administered 30 minutes after a meal for additional control. The only drawback is that substantial doses are needed compared to subcutaneous injections, ruling it out as a realistic alternative, at least in its current form. Larger doses would also mean a 5-10 times higher cost than conventional therapy to the patient, a problem that may be circumvented using the new once a week dispenser. With its somewhat poor bioavailability and amputated ability to normalize glucose levels on its own, clinicians have instead proposed using intranasal therapy as an additive to NPH insulin rather than a replacement, particularly after meals [28,29].

Moreover, this dependency on a significant basal insulin level may contribute to Type 1 DM patients [27]. Here, intranasal insulin was found to reduce blood sugar levels, an effect that lasted at least 4 hours without developing hypoglycemia [30]. This indicates that long term insulin would be needed for basal control. In contrast, regular subcutaneous insulin was found to have a delayed onset, substantial variability from person to person and daily [30]. Additionally, intranasal insulin is well-tolerated, decreasing T cell response to insulin [31]. It is also proposed as a long-term additive, not a replacement to subcutaneous insulin injections for the same reason.

Cognitive benefits and potential use of intranasal Insulin

Many animal and human studies have shown that enhanced insulin activity in the brain improves memory functions, especially declarative memory, which is hippocampus-dependent. Decreased permeation of blood-borne insulin across the blood-brain barrier and decreased signalling in the CNS are associated with Alzheimer's disease. Why not then overcome this CNS insulin deficiency by exogenously administering insulin? One therapeutic strategy uses the intranasal route, which can deliver insulin directly to the brain bypassing the blood-brain barrier. On these lines, Benedict et al. in 2010 demonstrated through a systematic review the benefits of intranasal insulin not only in improving memory of healthy people as well as of those with impaired cognition (like Alzheimer's Disease), in the absence of adverse effects, but also as a neuroprotective agent that may be used for prevention and treatment of insulin resistance and deficiency in the CNS as seen in Alzheimer's disease[32].

The effect of intranasally administered insulin on the CNS and the metabolic homeostasis of our body has also been studied in a review conducted by Ott et al. in 2012, wherein impaired insulin signalling in the brain was attributed to the development of type 2 diabetes and obesity, in which peripheral and possibly CNS insulin resistance is the hallmark, and Alzheimer's Disease, which has also been associated with insulin resistance as a contributor to the cognitive decline accompanying it [16].

After finding out about successful trials showing promising effects of intranasal intermediate-acting regular insulin for Mild Cognitive Impairment and Alzheimer's Disease, Claxton et al. performed an RCT to investigate whether the long-acting insulin detemir improves cognition in adults with these disorders. The trial was conducted on sixty adults with a diagnosis of either Mild Cognitive Impairment or mild/moderate Alzheimer's Disease, out of which 20 participants received placebo, 21 participants received insulin detemir (20 IU), and 19 participants received insulin detemir (40 IU) via a nasal drug delivery device, for 21 days. Results unravelled promising effects of 40 IU of insulin detemir administered intranasally in improving visuospatial (p<0.04) and verbal working memory (p<0.03). However, there were no significant differences seen in daily or executive functioning. APOE status (p<0.05) was found to moderate this effect, with APOE- ε4 carriers reflecting improvement while non-carriers were reflecting worsening of memory function, thereby suggesting that daily treatment with high dose (40 IU) intranasal insulin detemir modulated cognition for MCI or AD affected adults, with APOE-related differences in response to therapy [33].

Research on animals performed by Maimaiti et al. in 2016 revealed that AHP (Ca2+ dependent hippocampal hyperpolarization), a neurophysiological marker of ageing which is increased in the memory-impaired ageing animals, maybe a novel cellular target of insulin in the brain and is responsible for the improved cognitive performance after therapy with insulin administered via intranasal route [34].

A study carried out on diabetic mice by Rodrigues et al. in 2017 concluded that non-invasive intranasal insulin rescued central growth and neurogenesis, and reversed central atrophy and tau hyperphosphorylation, thus abrogating central pathology and cognitive decline in diabetic mothers' offspring. This opens the door to further research and individualized treatment alternatives for children exposed to poorly controlled gestational diabetes [35].

Avgerinos et al. published a systematic review in 2018 concluding that in patients with Mild Cognitive Impairment or Alzheimer's Disease, APOE4 (-), story recall performance was improved after receiving insulin intranasally. However, other cognitive functions were unaffected, but some positive results were noted in daily activity and functional status [36].

Research performed on 36 healthy men by Ritze et al. in 2018 demonstrated that long-term catabolic effects of CNS insulin delivery necessitate repetitive, preferably pre-meal insulin delivery schedules. Cognitive and memory improvement (delayed recall of words) is enhanced by sleep after intranasal administration of insulin. These findings were also associated with a decline in serum cortisol levels after two weeks of morning insulin administration. However, intranasal insulin administration did not induce any discernible changes in body weight and composition [37].

A 4-month long placebo-controlled clinical trial of 20 or 40 IU intranasal insulin published by Mustapic et al. in 2019 on Alzheimer's Disease patients to demonstrate biomarkers of insulin resistance and the treatment-associated changes and their relationship with cognitive performance (ADAS-Cog) concluded that neuronal enriched extracellular vesicle biomarkers of insulin resistance (pS312-IRS-1, pY-IRS-1) were associated with cognitive changes (ADAS-Cog) in response to low dose (20 IU) intranasal insulin, especially in APOE- ɛ4 non-carriers, suggesting insulin cascade involvement in neurons of genesis [38].

Intranasal insulin has also been found to provide functional improvement in Parkinson's disease and multiple system atrophy patients, according to this double-blinded placebo-controlled pilot study, conducted by Novak et al. in 2019, on these patients to evaluate the effects of 40 IU insulin or saline OD for four weeks on cognitive and functional performance [39].

Despite multiple studies confirming the benefits of intranasal insulin on cognition and memory, a Randomized Clinical Trial to investigate the safety, efficacy, and feasibility of intranasal insulin for the treatment of Mild Cognitive Impairment and Alzheimer's Disease, published by Craft et al. in 2020, revealed that intranasal insulin therapy given for 12 months had no cognitive or functional benefits among the primary intention-to-treat cohort with either Alzheimer's Disease or Mild Cognitive Impairment [40].

Intranasal vs Parenteral insulin administration

Oral drug therapy has always been the most preferred route of administration, especially in patients requiring chronic therapy. However, it is not always practical for the systemic delivery of certain drugs like insulin due to the 'first pass' metabolism and their clearance by both the liver and gastric mucosa, leading to the low bioavailability of drugs [2,41-44]. As a result of this, insulin is administered parenterally via the subcutaneous route. Subcutaneous insulin injections of either single or multiple doses have become the mainstay route of administration to deliver insulin therapy [24]. 

Although insulin can also be delivered by intravenous route or a continuous infusion using a portable infusion pump, their use is limited as it is not feasible for the patients who require a daily injection regimen. Even though the subcutaneous route is preferred over the other types of parenteral routes, it still has its own set of disadvantages, abysmal patient compliance due to the pain or discomfort while self-injecting and other side effects oedema, hypoglycemia, weight gain and lipodystrophy [45,46]. As the insulin absorption via the subcutaneous route is slow and sustained, it does not resemble the physiological "pulsatile "patterns of endogenous insulin secretion, which could result in hypoglycemic episodes despite the strict regulation of dose and site of injection [2,47-48]. These difficulties led to the investigation of alternate non-parenteral routes for insulin delivery.

 Many other non-parenteral routes like transdermal, buccal, ocular, pulmonary, nasal, vaginal and rectal routes have been investigated, but the intranasal route has been considered the most practical and favourable route for chronic drug therapies [13,49,50]. It is a rapid, non-invasive and easy method of administering insulin.

Systemic drug delivery via the intranasal route has been studied extensively to deliver compounds like peptides and proteins by this route [51,52]. Self-medication using this route has better patient compliance than parenteral routes as there is no associated pain or discomfort [46]. Due to the large absorptive surface area of the nasal cavity and the rich blood supply network in the nasal mucosa, the insulin rapidly enters the systemic circulation bypassing the first-pass metabolism altogether [51,53]. The pharmacokinetics of intranasal insulin is similar to that of intravenous insulin administration [51]. Intranasal insulin therapy does not result in hypoglycemic episodes in patients because it mimics the physiological pulsatile endogenous insulin secretion during meal times. Insulin, when given intranasally, also bypasses the blood-brain barrier [54-55] and enters the CNS without causing peripheral side effects [56-57], unlike parenterally given insulin.

 In conclusion, the low risk of systemic hypoglycemia and a better CNS absorption has prompted researchers to study the use of intranasal insulin therapy in the treatment of cognitive disorders such as Alzheimer's instead of the commonly used subcutaneous route or an IV route.

Adverse effects and limitations of intranasal Insulin

Adverse Effects/ Safety Profile

Even though intranasal insulin is well tolerated by most individuals [58], it can cause minor adverse effects. In various studies, adverse effects related to the nose(rhinitis, minimal nose bleed, soreness, dripping, sneezing), upper respiratory tract infections, headaches, dizziness, weakness, hypoglycemia, fall, rash, gastrointestinal symptoms are reported [36]. Rhinitis followed by other local side effects (nose related symptoms) is most commonly reported, which could be due to the mode of drug administration. The risk of hypoglycemia which is a significant concern with insulin, is negligible with the intranasal route [59].

Limitations

Although intranasal insulin has a better safety profile, the most significant limitation is the bioavailability of the drug, which is less than 1% through nasal administration, contributing by the low permeability of the nasal mucosa, rapid mucociliary clearance of the administered drug and the possible enzymatic degradation of the drug [42,60-62]. The low permeability is primarily due to the large molecular size of insulin [53]. The nasal cavity aminopeptidases (proteolytic enzymes) are the most common enzymes responsible for the degradation. The exopeptidases and the endopeptidases cleave the peptides at N, C terminals and the internal peptide bonds, respectively [42]. However, In a review, Duan and Mao described various new strategies to improve the intranasal absorption of insulin. The nasal absorption or transport of insulin can be improved by using water-insoluble powders with nasal absorption enhancers. The mucociliary clearance can be prevented by using mucoadhesive /bioadhesive drug delivery systems. The proteolytic degradation of the drug can be prevented by the chemical modification of the proteins, using proteolytic enzymes degraders [53]. However, these adsorption enhancers, bioadhesive and water-insoluble excipients should be screened furthermore for the safety profile and the effectiveness in increasing the nasal bioavailability of insulin [53].

## Conclusions

This literature review focused on the efficacy, benefits, and limitations of using Intranasal Insulin and its effect on the cognitive function of people with diabetes. Among the various anti-diabetic medications available, intranasal insulin appears to be the most effective means of increasing brain insulin levels, decreasing insulin resistance, a primary problem for many patients suffering from Diabetes Mellitus. Intranasal insulin delivery also avoids the negative consequences of subcutaneous insulin injections, bypassing peripheral circulation. People with diabetes may benefit from IN insulin if a long-acting version is administered instead of a short-acting form. Current findings are inconclusive as to whether IN insulin can solely treat dementia caused by Alzheimer's disease or MCI in people with diabetes. However, they give good evidence for its safety, as systemic adverse effects, such as hypoglycemia, were practically non-existent. Many pieces of research have shown that there have been improvements in visual-spatial memory and cognitive function among people with diabetes with pre-existing MCI or Alzheimers, likely due to its ability to cross the blood-brain barrier. We recommend that future trials be done with greater doses of IN Insulin and after careful selection of individuals and insulin types since this could be a potential breakthrough in neuroendocrinology.
